# Monitoring SARS-CoV-2 infection in urban and peri-urban wildlife species from Catalonia (Spain)

**DOI:** 10.1186/s42522-024-00109-5

**Published:** 2024-09-01

**Authors:** Leira Fernández-Bastit, Tomás Montalvo, Sandra Franco, Laura Barahona, Manel López-Bejar, Annais Carbajal, Encarna Casas-Díaz, Francesc Closa-Sebastià, Joaquim Segalés, Júlia Vergara-Alert

**Affiliations:** 1 Unitat Mixta d’investigació IRTA-UAB en Sanitat Animal, Centre de Recerca en Sanitat Animal (CReSA), Universitat Autònoma de Barcelona (UAB), Campus, Bellaterra, 08193 Catalonia Spain; 2 Programa de Sanitat Animal, IRTA, Centre de Recerca en Sanitat Animal (CReSA), Universitat Autònoma de Barcelona (UAB), Campus, Bellaterra, 08193 Catalonia Spain; 3 Agència de Salut Pública de Barcelona, Barcelona, Spain; 4 CIBER de Epidemiología y Salud Pública, CIBERESP, Madrid, Spain; 5 Institut de Recerca Sant Pau (IR SANT PAU), Sant Quintí 77-79, Barcelona, 08041 Spain; 6 Departament de Sanitat i Anatomia Animals, Facultat de Veterinària, Universitat Autònoma de Barcelona, Bellaterra, 08193 Catalonia Spain; 7 CLOSA IGM SL, Vets&Wildlife©, Terrassa, 08224 Catalonia Spain

**Keywords:** SARS-CoV-2, Surveillance, Urban species, Peri-urban species, Rodents, wild boar

## Abstract

**Background:**

Human activities including deforestation, urbanization, and wildlife exploitation increase the risk of transmission of zoonotic diseases. Urban and peri-urban wildlife species often flourish in human-altered environments, with their survival and behavior heavily influenced by human-generated food and waste. In Catalonia, Spain, and other Mediterranean regions, species of rodents, including the house mouse (*Mus musculus)*, black rat (*Rattus rattus)*, Norway rat (*Rattus norvegicus)*, as well as wild boar (*Sus scrofa*) are common in urban and peri-urban areas. These species host numerous infectious agents, including coronaviruses (CoVs), posing potential human health risks. During the coronavirus disease 2019 (COVID-19) pandemic, the severe acute respiratory syndrome coronavirus 2 (SARS-CoV-2) evolved to infect previously non-susceptible species, with variants capable of infecting rodents, emphasizing their importance in surveillance studies.

**Methods:**

The present study assessed SARS-CoV-2 presence and/or exposure in 232 rodents, 313 wild boar, and 37 Vietnamese Pot-bellied pigs in Catalonia during the pandemic period (2020–2023).

**Results:**

All the animals tested for acute SARS-CoV-2 infection (232 rodents and 29 wild boar) were negative. For SARS-CoV-2 exposure, 3 out of 313 (0.96%) wild boar tested positive by ELISA, while the remaining 32 rodents, 310 wild boar, and 37 Vietnamese Pot-bellied pigs were all negative. Cross-reactivity with other CoVs was predicted for ELISA-positive samples, as the 3 wild boar tested negative by the virus neutralization assay, considered as the gold standard technique.

**Conclusions:**

The absence of SARS-CoV-2 exposure or acute infection in wild boar and rodent species supports their negligible role in viral spread or transmission during the COVID-19 pandemic in Catalonia. However, their proximity to humans and the ongoing genetic evolution of SARS-CoV-2 underline the need for continued monitoring. Surveillance of SARS-CoV-2 infection in animal species can contribute to design measures to control the emergence of new animal reservoirs or intermediate hosts that could facilitate viral spillover events.

## Introduction

Urban or peri-urban species refer to animals found in urban environments or in the transition area between urban and rural worlds, respectively [1]. These species are heavily influenced by human activities and often thrive in human-altered environments such as parks, gardens, agricultural, industrial areas, and even buildings [2]. They may display adaptive behaviors, such as foraging in garbage bins filled with human-produced waste or nesting in man-made structures. The impact of urban and peri-urban species on humans can vary widely and depends on the specific species and the interaction with the human environment. Regarding health considerations, these species may play a role in the transmission of zoonotic diseases to humans, especially if they act as reservoirs and if there is a close animal-human contact [2].

In Catalonia, Spain, and on a global scale, rodent species including the house mouse (*Mus musculus*; *MM*), black rat (*Rattus rattus*; *RR*), and Norway rat (*Rattus norvegicus; RN*), are recognized as urban pest species [3–5]. Generally, *MM* are predominantly found indoors, particularly in buildings and homes, while *RN* are commonly sighted in sewers, garbage areas, and buildings [3, 5]. Besides, *RR* are well adapted to naturalized environments, thriving in parks and green areas. Considering that rodents are carriers of at least 60 zoonotic diseases, their proximity to humans may pose a substantial threat to human health [6, 7]. Accordingly, *alpha-* and *beta-coronaviruses* have been identified in these animal species in China and Europe [7–10]. Indeed, both HCoV-OC43 and HCoV-KU1 are human coronaviruses (CoVs) that have a rodent origin, underlining the potential role of these animals in disease transmission [11]. At the outset of the coronavirus disease 2019 (COVID-19) pandemic, rodents were initially considered non-susceptible to severe acute respiratory syndrome coronavirus 2 (SARS-CoV-2) [12]. However, the ongoing genetic evolution of the virus triggered the emergence of various viral variants capable of infecting these rodent species [12–15].

On the other hand, wild boar (*Sus scrofa*) represent a notable example of peri-urban species in Catalonia and most of Europe [16]. Catalonia has a significant population of wild boar, and its presence is influenced by factors including habitat availability, food resources, and human activity. Currently, this animal is predominantly found in North-Eastern Catalonia and the province of Barcelona, with population densities ranging from 9 to 15 individuals/km^2^ [16]. Expansive urban and agricultural areas, along with abundant vegetation, provide favorable conditions for the population to grow and thrive [16]. Estimates of wild boar density in monitoring programs are determined based on hunting captures in naturalized environments [16]. The invasive population of this species causes significant impacts on local ecosystems, potentially contributing to the spread of diseases that affect both wildlife and domestic animals, and even posing risks to human health [17, 18]. Wild boar can transmit diverse zoonotic diseases to humans including Hepatitis E, brucellosis, salmonellosis, tuberculosis, yersinosis, toxoplasmosis and trichinellosis [18]. Additionally, the Vietnamese Pot-Bellied pig (*Sus scrofa domestica*) is a small-sized domestic breed that gained popularity as a pet breed in various parts of the world due to its friendly temperament. Importantly, domestic pigs can be infected by six different CoVs, but not SARS-CoV-2, at least the ancestral variant upon experimental infection [19–21]. However, the susceptibility of pigs to subsequently emerged variants has not been assessed experimentally. Considering that many variants have expanded their host range [12, 15], assessing the exposure to these variants of SARS-CoV-2 in this animal species should not be ignored.

Therefore, this study aimed to monitor evidence of exposure to and/or acute infection by SARS-CoV-2 in rodent species and wild boar, as well as Vietnamese Pot-bellied pigs, found in Catalonia. The study used samples from the entire COVID-19 pandemic period (from 2020 to 2023) to encompass exposure to all the different SARS-CoV-2 variants that emerged within the study area.

## Materials and methods

### Samples

**Fig. 1:**
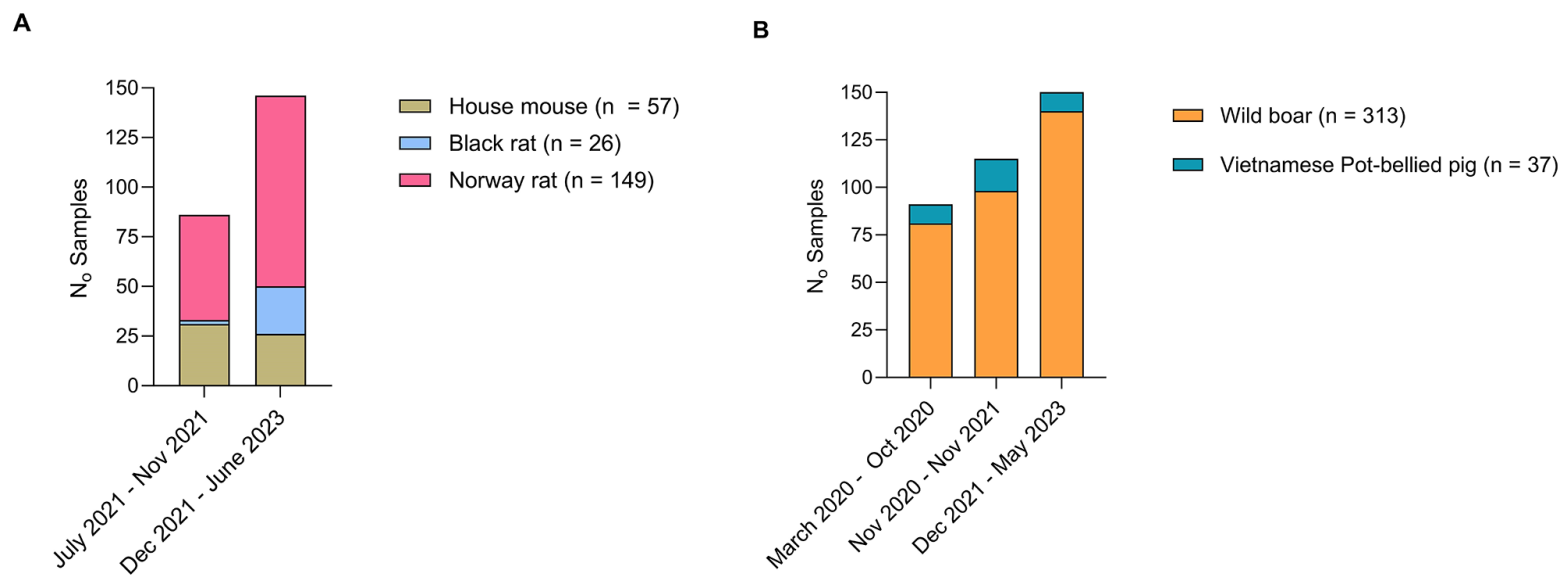
Animal sampling in Catalonia during the COVID-19 outbreak with respect to the SARS-CoV-2 variants [22]. **A**) Rodent species sampling distribution in two phases: Firstly, running between July and November 2021 predominantly for Alpha (B.1.1.7) and Delta (B.1.617.2) variants of SARS-CoV-2. Next, from December 2021 to June 2023 predominantly for the Omicron variant and its subvariants. B) Wild boar and Vietnamese Pot-bellied pigs sampling distribution across three phases: Firstly, running between March 2020 and October 2020 predominantly for the ancestral variant (B.1). Next, between November 2020 and November 2021 predominantly for the Alpha (B.1.1.7) and Delta (B.1.617.2) variants, and lastly, between December 2021 and May 2023 predominantly for Omicron and its subvariants

This study included a total number of 582 animals of which 232 were rodents (precisely 57 *MM*, 26 *RR* and 149 *RN*), 313 wild boar, and 37 Vietnamese Pot-bellied pigs **(**Table 1**).** Samples were collected opportunistically between July 2021 and June 2023 for rodent species, and between March 2020 and May 2023 for both wild boar and Vietnamese Pot-bellied pigs **(**Fig. 1**).**

Oral swabs (57 *MM*, 24 *RR* and 148 *RN*), lung tissues (57 *MM*, 26 *RR* and 136 *RN*), and sera (21 *RR* and 11 *RN*) were collected from rodents. At least a sample type was obtained from each rodent **(**Table 1**).** On the other hand, oral swabs were collected from 29 out of 313 wild boar and serum samples were collected from all wild boar (*n* = 313) and all Vietnamese Pot-bellied pigs (*n* = 37) **(**Table 1**).** The types of samples collected from each animal species were determined by both availability and the challenges of obtaining samples.

Oral swabs were taken using sterile dry swabs or DeltaSwab ViCUM^®^ contained in 2 mL viral transport media (VTM) (Delta-lab, S.L., Catalonia, Spain). As for lung tissue samples, approximately 0.2 mg was placed into cryotubes containing 500 µL of Dulbecco’s Modified Eagle Medium (DMEM) (Lonza, Basel, Switzerland) supplemented with 100 U/mL penicillin, 100 µg/mL streptomycin, and 2 mM glutamine (all from Gibco Life Technologies, Madrid, Spain) with a single 4.5-mm, zinc-plated steel bead.

**Fig. 2:**
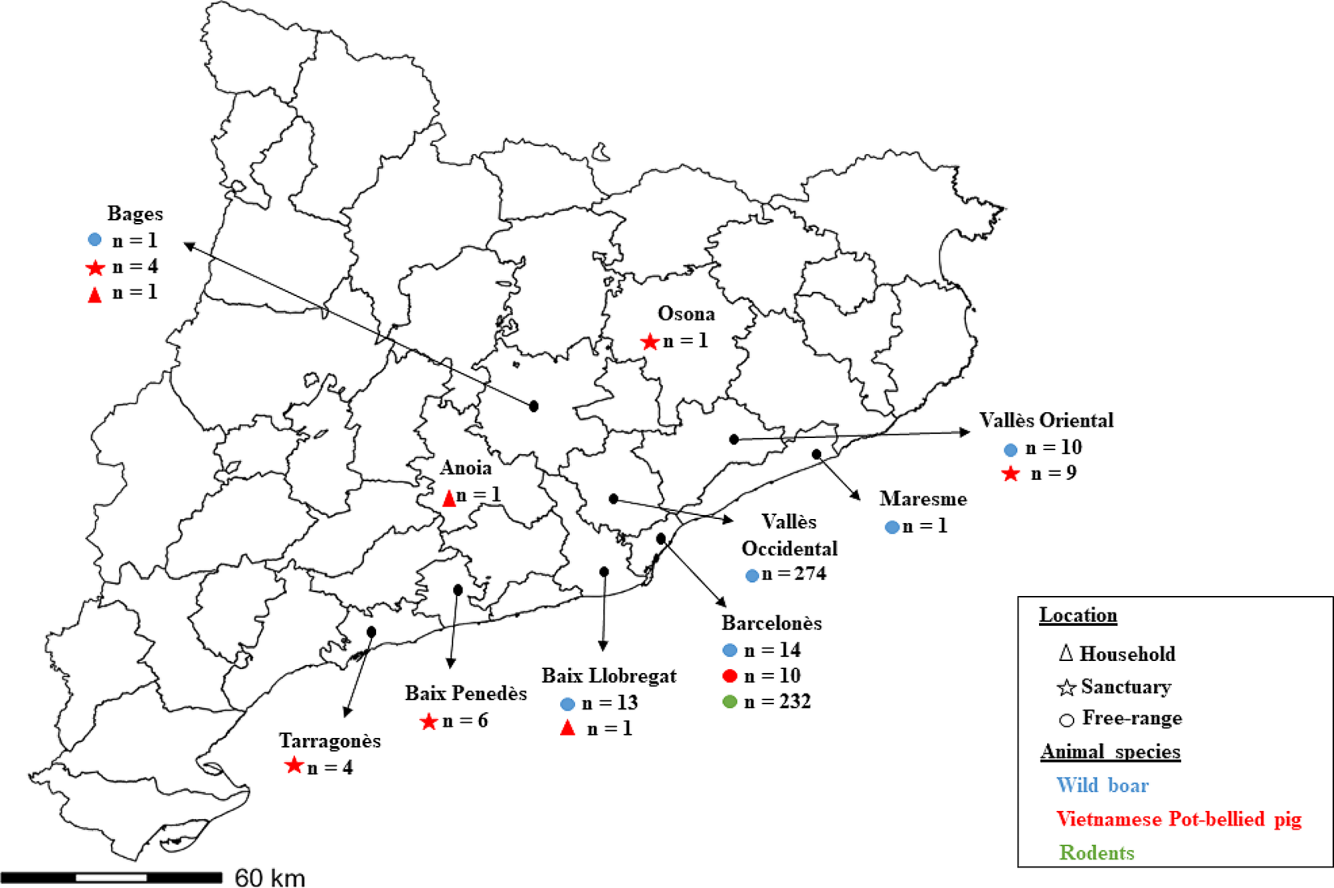
Map illustrating the geographical distribution of sampled animals across counties in Catalonia during the COVID 19 pandemic. Wild boar (*n* = 313) are denoted in blue, Vietnamese Pot-bellied pigs (*n* = 37) in red, and rodents in green (*n* = 232). Triangles represent animals ourced from households, stars indicate animals from sanctuaries, and circles signify free-ranging animals from urban and peri-urban areas

The trapping of rodent species (*MM*, *RR* and *RN*) was conducted in the city of Barcelona (Catalonia, Spain) **(**Fig. 2**)** by the *Agència de Salut Pública de Barcelona* (ASPB), which is the authority responsible for pest surveillance and control in Barcelona. For *MM*, the trapping was carried out in municipal facilities (e.g., libraries, civic centers, retirement homes, municipal markets) **(**Table 1**).** Addressing pest complaints and mice infestation is the major goal of the pest surveillance program. In locations where mice activity was detected, live capture traps were installed. Traps were checked every two days: traps with signs of activity were left in place; traps with no signs of mice activity were removed after a week. Rats samples were obtained from individuals captured during studies conducted in the sewage system (137/149 *RN*) and within public green areas of the city (12/149 *RN* and 26 *RR*) **(**Table 1**).** Rats in the sewers were captured with snap traps, while live traps were used in public green areas.

On the other hand, wild boar and 10 out of the 37 (27.03%) Vietnamese Pot-bellied pigs were captured with traps located in urban and peri-urban areas from different counties of Barcelona **(**Fig. 2**).** Besides, from the 27 Vietnamese Pot-bellied pigs that were not free-ranging, 14 (51.85%) and 10 (37.04%) came from sanctuaries in the province of Barcelona and Tarragona (Catalonia, Spain), respectively, and three (11.11%) were from separate households from the province of Barcelona **(**Fig. 2**).** Sampling of wild boar and free-range Vietnamese Pot-bellied pigs was performed by trap capture and anesthesia. The cage traps were 1.03 m in length, 1 m in width, and 1.48 m in height and were made from welded rods. These rods, with a diameter of 8 to 10 mm, formed a mesh with squares measuring 5 cm x 5 cm. They featured downward-opening doors activated by a trigger mechanism wired to these doors by steel cables. The traps were baited with corn and checked daily. The animals were kept in the cage traps for an average period of 12 h (range 8–16 h) before blood collection. In order to minimize stress, animal handling took place during the early morning hours, and anesthesia was administered by a single person approaching the animal. Animals were anesthetized using a combination of tiletamine–zolazepam (6 mg/kg, Zoletil Virbac Salud Animal, Esplugues de Llobregat, Spain) and xylazine (3 mg/kg, Xilagesic 20%, Calier Laboratories, Les Franqueses del Vallès, Spain), delivered via a dart syringe dispatched with a blowpipe (Telinject, Global Veterinaria, Mataró, Spain). Once anesthetized, the animals were placed in lateral recumbency and blood samples were collected from the heart using 18 G 1½″ disposable needles (Sterican; Bbraun, Rubí, Spain) and 10 mL syringes (Omnifix; Bbraun). Euthanasia was then performed by same the administration methods (1 mL/10 kg, Euthasol, Dechra Veterinary Products SLU, Barcelona, Catalonia, Spain). Vietnamese Pot-bellied pigs from households were anesthetized in a similar manner to the free-range wild boar.

category). Zoos and rehabilitation centers are represented by letters (A N) and the animal source (free ranging or zoo) is indicated by figures (triangle and square, respectively). Positive results in diagnostic tests are in.

**Table 1:** Population sizes of each animal species categorized by locations, along with the types and quantities of samples (oral swabs, lung tissues, and serum samples) obtained from each species. NA: non-available

Animal species	Location	*n*	Oral swabs	Lung tissue	Serum sample
House mouse *(Mus musculus)*	Municipal facilities	**57**	57	57	NA
Black rat (*Rattus rattus*)	Green areas	**26**	**24**	**26**	**21**
Norway rat (*Rattus norvegicus*)	Sewage system	137	136	124	NA
	Green areas	12	12	12	11
	**Total**	**149**	**148**	**136**	**11**
Wild boar (*Sus scrofa*)	Free-range	**313**	**29**	**NA**	**313**
Vietnamese Pot-bellied pig (*Sus scrofa domesticus*)	Free-range	10	NA	NA	10
	Sanctuaries	24	NA	NA	24
	Households	3	NA	NA	3
	**Total**	**37**	**NA**	**NA**	**37**

### RNA extraction and detection of SARS-CoV-2 by RT-qPCR

All rodents (*n* = 232; 57 *MM*, 26 *RR* and 149 *RN*) and 29 out of the 313 wild boar were tested for acute infection of SARS-CoV-2. The presence of SARS-CoV-2 RNA in oral swabs and/or lung tissue samples was assessed by reverse transcriptase-quantitative PCR (RT-qPCR). Sterile dry swabs were transferred into cryotubes containing 500 µL supplemented DMEM and finally vortexed. DeltaSwabs ViCUM^®^ with VTM were directly vortexed. Lung tissue samples were mechanically homogenized at 30 Hz for 1 min using a TissuLyser II (QIAGEN GmbH, Hilden, Germany) and centrifuged for 3 min at 10,000 rpm. Then, all samples were subjected to RNA extraction according to the manufacturer’s instructions of the Indimag Pathogen Kit (Indical Biosciences Leipzig, Germany) and using a Biosprint 96 workstation (Qiagen, Hilden, Germany). SARS-CoV-2 RNA detection was carried out using a previously described protocol targeting the envelope protein (E)-encoding gene [23] with minor modifications [24]. Briefly, RT-qPCR was performed using the AgPath-ID TM One-Step RT-PCR Reagents (Applied Biosystems, Life Technologies, Waltham, MA, USA) and amplification was done using a 7500 Fast Real-Time PCR System (Applied Biosystems, Life Technologies, Waltham, MA, USA). Cq values < 40 indicated a positive result for SARS-CoV-2 RNA detection [23].

### Detection of SARS-CoV-2 antibodies

Blood samples (*n* = 382; 21 *RR*, 11 *RN*, 313 wild boar and 37 Vietnamese Pot-bellied) were used to test the exposure to SARS-CoV-2 by detecting neutralizing antibodies (nAbs) against the receptor binding domain (RBD). First, blood samples were centrifuged at 1800 x g for 10 min at 4 °C, and the resulting sera were then inactivated at 56 °C for 30 min. The assessment of nAbs against the SARS-CoV-2 RBD was performed using the Genscript cPass ^TM^ SARS-CoV-2 Neutralization Antibody Detection Kit (Genscript, the Netherlands), following the manufacturer’s protocol. The percentage of inhibition of the RBD-ACE2 interaction was calculated using the following formula: % Inhibition = (1 – (OD450 sample/OD450 negative control)) × 100. Samples with an inhibition proportion of ≥ 30% were considered positive for presence of SARS-CoV-2 RBD nAbs.

ELISA-positive samples were further analyzed by a virus neutralization test (VNT) as previously described [25]. Briefly, sera were diluted 1/10 and then 2-fold serially diluted in supplemented DMEM. These were mixed 1:1 with a SARS-CoV-2 isolate (D614G strain) from a COVID-19 patient (GISAID ID EPI ISL 471,472). After 1 h of incubation at 37 °C, each dilution mixture (in duplicates) was transferred to Vero E6 monolayers containing 100 Tissue Culture Infectious Dose 50 (TCID_50_) of SARS-CoV-2 per well and cultured for 3 days at 37 °C and 5% CO_2_. Then, the CellTiter-Glo luminescent cell viability assay (Promega, Madison, WI, USA) was performed in accordance with the manufacturer’s protocol to measure the cytopathic effect (CPE). Luminescence was measured as luminescence units in a Fluoroskan Ascent FL luminometer (ThermoFisher Scientific, Waltham, MA, USA). The serum virus neutralization titer 50 (SNT_50_) corresponds to the reciprocal value of the sample dilution showing 50% of the SARS-CoV-2-induced CPE in Vero E6 cells.

Seroprevalence and 95% confidence intervals were calculated in each population.

### Ethical approval

Permission to carry out the study of rodent species was granted by the Department of Territory and Sustainability of the regional government of Catalonia (reference number: SF/044). Rats were treated according to Directive 2010/63/EU of the European Parliament and Council decision of 22nd September 2010 concerning the protection of animals used for scientific purposes.

Wild boar were captured according to the requirements and permissions issued by the Department of Climate Action, Food and Rural Agenda of the Autonomous Government of Catalonia (EPI-53/2019, EPI-29/2021, AC/259 − 20 and AC/292 − 21).

## Results

All animal species tested for the presence of SARS-CoV-2 RNA by RT-qPCR (232 rodents and 29 wild boar) were negative (Ct ≥ 40).

As assessed with the blocking RBD-ELISA test, three out of the 313 (0.96%; CI: 0.0-2.06%) wild boar tested positive for the presence of nAbs against the RBD with a low percentage of inhibition in each sample: 35.22% (Wild boar 1 – Wb1), 34.87% (Wild boar 2 – Wb2) and 30.20% (Wild boar 3 – Wb3). Wb1 was sampled in April 2020, Wb2 in May 2021, and Wb3 in August 2021. ELISA-positive sera samples were subsequently tested by VNT, the gold standard technique to confirm specific viral neutralization, and all tested negative. The remaining 310 wild boar, 21 *RR* and 11 *RN*, tested negative by ELISA and were not subjected to further VNT testing.

## Discussion

Previous events of reverse zoonotic transmission of SARS-CoV-2 and the virus’s ability to adapt and spread in some animal species, have underscored the need for surveillance studies in species at risk of infection. Initially, murine and wild boar were not deemed susceptible to SARS-CoV-2. However, variants of SARS-CoV-2 that emerged during the pandemic demonstrated their potential to infect a wider host range as well as previously non-susceptible, such as rats and mice [12, 15, 26]. As a result, this study aimed to assess the presence of acute infection or exposure to SARS-CoV-2 throughout the entire pandemic period (2020–2023) in rodents and wild boar for better understand the prevalence and distribution of the disease in urban and peri-urban wildlife populations.

Essentially, the results revealed that none of the animals included in this study, whether rodents or wild boar, had an acute SARS-CoV-2 infection at the time of sampling, as negative results were observed by RT-qPCR. Additionally, serological analyses indicated that none of the animals had been exposed to the virus, as no specific nAbs were detected in blood samples. Initial serological screening using the RBD-inhibition ELISA assay revealed that three wild boar out of 313 had nAbs against SARS-CoV-2. Nevertheless, these animals tested negative by VNT, a more specific and reliable technique, suggesting potential false positives in the ELISA results. Six different CoVs (four *alphacoronaviruses*, one *betacoronavirus* and one *deltacoronavirus*) are known to infect pigs [27, 28], and a certain degree of cross-reactivity between antibodies for these and SARS-CoV-2 has already been proposed [29].

At the onset of the COVID-19 pandemic, experimental infections demonstrated that domestic pigs were not susceptible to the ancestral variant of SARS-CoV-2 by intranasal, intratracheal (IT), intramuscular (IM) or intravenous (IV) routes of inoculation [19–21, 30]. However, when piglets were parenterally inoculated (IM and IV), antibodies against the spike (S) glycoprotein were observed at least 14 days post inoculation (dpi) and nAbs were detected at 22 dpi [20]. Notably, the inoculation doses (≈ 10^5^ − 10^6^TCID_50_/mL) in most studies on pig susceptibility were likely higher [19–21] than what a host encounters naturally. Besides, in vitro studies demonstrated that SARS-CoV-2 can replicate and cause CPE in porcine cell lines, including swine testicle and porcine kidney cells (PK-15) [31, 32]. Accordingly, the expression of the angiotensin converting enzyme 2 (ACE2), the primary cell receptor for SARS-CoV-2, has been verified in pig intestine and kidneys, contrasting with its absence in the respiratory tract (RT) [33]. Since SARS-CoV-2 mainly utilizes the RT as infection entry point, the risk of infection in pigs and wild boar in natural conditions might be considered low. Nonetheless, wild boar’s urban behavior, proximity to human populations, and interaction with human-produced waste justify their inclusion in monitoring studies to assess viral exposure. Additionally, the possibility of alternative virus receptor enabling infection in specific species cannot be ruled out [34, 35].

On the other hand, we examined the exposure to SARS-CoV-2 in *21* RR and *11* RN, with all individuals testing negative for SARS-CoV-2 RBD nAbs. However, the limited number of serum samples from this group of animals may restrict the generalizability of our findings to the entire population. Consequently, our results do not conclusively rule out the possibility of SARS-CoV-2 exposure in rodents in Barcelona during the pandemic. *RN* are likely thriving in the sewer environment where they have access to food and water. Notably, SARS-CoV-2 has been detected in wastewater from the sewer system of various countries due to virus particles in feces and urine from infected humans [36, 37]. Indeed, detection of SARS-CoV-2 RNA in wastewater has been utilized in epidemiological studies to determine SARS-CoV-2 incidence and predict the emergence of novel variants in the human population in Catalonia [36]. However, the absence of evidence of infectious virus in wastewater or fecal waste significantly reduces the risk of infection among animals [38]. Consistent with our findings, a study on SARS-CoV-2 surveillance in *RN* within the Antwerp, Belgium, sewage system also reported a lack of exposure to the virus, as they did not detect SARS-CoV-2 nAbs [39]. In contrast, Wang et al. (2023) proposed that this species may have been exposed to SARS-CoV-2 in the sewage system of New York City, based on ELISA testing [40]. However, the conflicting negative results obtained from the microneutralization assay casted doubt on this assertion [40]. The same authors also identified partial genomic sequences of SARS-CoV-2 (with coverage ranging from 1.6 to 21.3%) associated with the B.1 lineage in four *RN*, two of which being from rats that tested positive in ELISA testing [40]. Another study performed in Liverpool, UK, also supported the possibility of SARS-CoV-2 exposure in this species in the sewer system, as antibodies in lung and heart tissue fluid partially neutralized pseudovirus particle infection [41]. Low titers of nAbs against SARS-CoV-2 were also found by VNT in one *RN* in Hong Kong (China) on May 2021, as part of a surveillance study in rodent species [42].

The need to monitor murine species arose as variants of SARS-CoV-2 gained the ability to infect them, contrarily to the ancestral variant (B.1) [12, 15]. Due to specific amino acid substitutions within the ACE2-RBD interacting surface on murine ACE2 (mACE2) compared with human ACE2, the SARS-CoV-2 ancestral variant was not able to use murine ACE2 for cell entry [12]. However, viral variants carrying the N501Y mutation in the RBD of SARS-CoV-2, including Alpha (B.1.1.7), Beta (B.1.351), Gamma (P.1) and Omicron (B.1.1.529), increased the ability of SARS-CoV-2 to bind mACE2 and thus infect murine species [12, 43]. Rodents are known to be suitable reservoirs of zoonotic diseases due to several factors that facilitate the transmission of pathogens to humans [6, 44]. These factors include rapid reproduction rates and their adaptability to diverse environments [6]. Rodents nesting close to human dwellings and feeding on stored food in homes or urban areas can contribute to the transmission of diseases [6]. Therefore, reducing the risk of SARS-CoV-2 variants spreading to these animals and understanding the potential role these species may play in transmission is crucial. Additionally, since rodent species host a range of CoVs, there is a the possibility for viral recombination, including the recombination of SARS-CoV-2 with other CoVs [10]. This could lead to the emergence of viral variants that pose a major risk for both human and animal well-being [10, 41].

## Conclusions

Findings from our study indicated that urban and peri-urban populations of wild boar and rodents in Catalonia reported no signs of exposure to or acute infection with SARS-CoV-2. This suggests that these species were unlikely to have played a role in spreading or transmitting the virus during the COVID-19 pandemic. However, the potential for new variants of SARS-CoV-2 to expand their host range underlines the importance of ongoing surveillance of these animal populations, especially rodent species. This is crucial due to their close contact with human communities, which could pose future risks of zoonotic transmission.

Wild boar were captured according to the requirements and permissions issued by the Department of Climate Action, Food and Rural Agenda of the Autonomous Government of Catalonia (EPI-53/2019, EPI-29/2021, AC/259 − 20 and AC/292 − 21).

## Data Availability

The datasets used and/or analysed during the current study are included in this published article.

## Abbreviations

ACE2: Angiotensin converting enzyme 2
CoVs: Coronaviruses
COVID-19: Coronavirus disease 19
CPE: Cytopathic effect
Dpi: Days post inoculation
ELISA: Enzyme-linked immunosorbent assay
IM: Intramuscular
IT: Intratracheal
IV: Intravenous
mACE2: Murine angiotensin converting enzyme 2
MM: Mus musculus
nAbs: Neutralizing antibodies
RBD: Receptor binding domain
RN: *Rattus norvegicus*
RR: *Rattus rattus*
RT: Respiratory tract
RT-qPCR: Reverse transcription – quantitative polymerase chain reaction
SARS-CoV-2: Severe acute respiratory syndrome coronavirus 2
SNT_50_: Serum virus neutralization titre 50
TCID_50_: Tissue Culture Infectious Dose 50
VNT: Virus neutralization test
Wb1: Wild boar 1
Wb2: Wild boar 2
Wb3: Wild boar 3
